# *Gelidium amansii* Attenuates Hypoxia/Reoxygenation-Induced Oxidative Injury in Primary Hippocampal Neurons through Suppressing GluN2B Expression

**DOI:** 10.3390/antiox9030223

**Published:** 2020-03-09

**Authors:** Md. Abdul Hannan, Md. Nazmul Haque, Md. Mohibbullah, Raju Dash, Yong-Ki Hong, Il Soo Moon

**Affiliations:** 1 Department of Anatomy, Dongguk University College of Medicine, Gyeongju 38066, Korea; hannanbmb@bau.edu.bd (M.A.H.); rajudash.bgctub@gmail.com (R.D.); 2 Department of Biochemistry and Molecular Biology, Bangladesh Agricultural University, Mymensingh-2202, Bangladesh; 3 Department of Fisheries Biology and Genetics, Patuakhali Science and Technology University, Patuakhali-8602, Bangladesh; habib.uni.ac.bd@gmail.com; 4 Department of Fishing and Post Harvest Technology, Sher-e-Bangla Agricultural University, Sher-e-Bangla Nagar, Dhaka-1207, Bangladesh; mmohib08@gmail.com; 5 Department of Biotechnology, Pukyong National University, Namku, Busan 608-737, Korea; ykhong@pknu.ac.kr

**Keywords:** oxidative stress, reactive oxygen species, hypoxia-reoxygenation, GluN2B, CNS neuron, *Gelidium amansii*

## Abstract

Oxidative stress is known to be critically implicated in the pathophysiology of several neurological disorders, including Alzheimer’s disease and ischemic stroke. The remarkable neurotrophic activity of *Gelidium amansii,* which has been reported consistently in a series of our previous studies, inspired us to investigate whether this popular agarophyte could protect against hypoxia/reoxygenation (H/R)-induced oxidative injury in hippocampal neurons. The primary culture of hippocampal neurons challenged with H/R suffered from a significant loss of cell survival, accompanied by apoptosis and necrosis, DNA damage, generation of reactive oxygen species (ROS), and dissipation of mitochondrial membrane potential (ΔΨ_m_), which were successfully attenuated when the neuronal cultures were preconditioned with ethanolic extract of *G. amansii* (GAE). GAE also attenuated an H/R-mediated increase of BAX and caspase 3 expressions while promoting Bcl-2 expression. Moreover, the expression of *N*-methyl-d-acetate receptor subunit 2B (GluN2B), an extrasynaptic glutamate receptor, was significantly repressed, while synaptic GluN2A expression was preserved in GAE-treated neurons as compared to those without GAE intervention. Together, this study demonstrates that GAE attenuated H/R-induced oxidative injury in hippocampal neurons through, at least in part, a potential neuroprotective mechanism that involves inhibition of GluN2B-mediated excitotoxicity and suppression of ROS production, and suggests that this edible seaweed could be a potential source of bioactive metabolites with therapeutic significance against oxidative stress-related neurodegeneration, including ischemic stroke and neurodegenerative diseases.

## 1. Introduction

Ischemia/reperfusion (I/R) installs a cascade of pathological events that culminate in several clinical conditions, including ischemic stroke and degenerative brain disease [1]. The I/R provokes a deleterious mechanism that involves excess production of reactive oxygen species (ROS), resulting in the oxidative stress in cells [2]. With high metabolic demand and peroxidizable lipid contents, neurons are potentially vulnerable to I/R-induced oxidative injury, which is accompanied by apoptosis, necrosis, and DNA damage. In reperfused cells, mitochondria play a critical role in initiating apoptosis through contributing excess ROS generation. During I/R, there is an involvement of glutamate excitotoxicity, to which the expression of extrasynaptic glutamate receptors such as GluN2B has been crucially implicated. This I/R-induced excitotoxicity initiates a cascade mechanism that results in the cellular oxidative stress and loss of mitochondrial membrane potential (ΔΨ_m_). Targeting excitotoxicity-mediated oxidative stress might, therefore, be a potential therapeutic strategy in neurodegenerative diseases.

A neuronal model of hypoxia/reoxygenation (H/R) provides a useful tool for the study of ROS-mediated mechanisms of cellular dysfunction in I/R injury in the brain [1]. In this study, a cellular model of H/R-induced oxidative injury that can simulate the pathophysiological events of cerebral I/R was, therefore, employed to investigate the neuroprotective effect of GAE in primary hippocampal neurons. In several of our previous studies, we reported a very potential neurotrophic activity of GAE that spans every stage of neuronal developments [3], including early neuronal differentiation, axonal sprouting, dendritic arborization, axonal maturation and synaptic modulation [4,5,6]. In addition, GAE has shown various other pharmacological activities, such as immunomodulation [7] and antioxidation [8]. Having all these pharmacological effects, we hypothesized that GAE could protect against H/R-induced oxidative injury in primary hippocampal neurons.

## 2. Materials and Methods

### 2.1. Sample Collection and Extract Preparation

The mature thalli of *G. amansii* were collected along the coast of the southern part of the Korean peninsula and processed as described in our previous study [4]. An ethanolic extract of *G. amansii* (GAE) was then prepared following the protocol as previously described [4]. The extract was reconstituted in dimethyl sulfoxide (DMSO) to make an aliquot of 8 mg/mL.

### 2.2. Primary Neuronal Culture and GAE Treatment

All the reagents used for cell cultures were purchased from Invitrogen (Carlsbad, CA, USA) unless otherwise stated. The animal experiment was approved by the Institutional Animal Care and Use Committee of the Dongguk University College of Medicine (approval certificate number IACUC-2016-001). Time-pregnant rats (Sprague-Dawley) were ordered on the 13th day of pregnancy and housed in controlled temperature with a light/dark cycle of 12/12 h and with access to food and water *ad libitum*. On the 19th day of pregnancy, the pregnant rat was euthanized with isofluorane, and the fetuses were collected. The dissociated cultures of primary hippocampal neurons from the fetal brain were prepared as previously described [9]. The dissected hippocampi were collected in Hank’s balanced salt solution (HBSS), and the tissues were dissociated by digestion with 0.25% trypsin in HBSS for 12 min at 37 °C and trituration with fire-polished graded Pasteur pipettes. The dissociated cells were seeded at a density of 3.0 × 10^4^ cells/cm^2^ onto poly-dl-lysine-coated (PDL, Sigma-Aldrich, St. Louis, MO, USA) 12-mm glass coverslips in 24-well culture plates for morphological and viability analysis, or 3.0 × 10^6^ cells/cm^2^ and 1.2 × 10^5^ cells/cm^2^ onto PDL-coated six-well culture plates for agarose gel electrophoresis and Western blot, respectively. Cultures were maintained in a defined serum-free neurobasal media supplemented with B27 and incubated at 37 °C under 5% CO_2_ and 95% air. The culture medium was preincubated with GAE or vehicle (DMSO, final concentration <0.5%). 

### 2.3. Hypoxia/Reoxygenation (H/R) Injury

Neurons were exposed to H/R following the protocol as described previously [10], with a slight modification. Briefly, at the indicated time, cultured neurons were transferred to a hypoxic chamber (Modular Incubator Chamber MIC-101; Billups-Rothenberg Inc., Del Mar, CA, USA) containing 94% N_2_, 5% CO_2_, and 1% O_2_ and incubated for 4 h at 37 °C. The culture plates were then returned to normoxic conditions (95% air and 5% CO_2_ at 37 °C) and incubated for the indicated time.

### 2.4. Assessment of Neuronal Viability and Cytotoxicity

*Trypan blue exclusion assay.* Neuronal viability was determined by trypan blue exclusion assay in cultures maintained in normoxic and hypoxic conditions. The cultures were stained with 0.4% trypan blue for 15 min at room temperature and then washed with Dulbecco’s phosphate-buffered saline (D-PBS). The neurons were then quantified under a microscope (Leica Microsystems AG, Wetzlar, Germany). Dead neurons are compromised to membrane permeability, and thus, uptake dye and appeared dark-blue in phase-contrast images. Live neurons have intact membrane integrity, and thus, exclude dye. The viability was expressed as the percentage of trypan blue-impermeable cells (live neurons) and results were normalized versus trypan blue-stained non-H/R exposed controls. In each experiment, cells on three coverslips, each with 500 cells, were counted randomly.

*Measurement of lactate dehydrogenase (LDH) release*. The cellular injury was evaluated by measuring the LDH released in the culture media through the damaged cell membrane using the CytoTox96 nonradioactive assay (Promega, Madison, WI, USA) and quantitated by measuring the wavelength at 490 nm using a microplate reader (Molecular Devices, San Jose, CA, USA). LDH activity is the percentage of the ratio of experimental LDH release with maximum LDH release. Data are normalized to the amount of LDH released from vehicle-treated cells (100%).

### 2.5. Measurement of Apoptotic Cell Death

Neurons that underwent apoptotic and necrotic death were determined by Annexin V binding and propidium iodide (PI) uptake, respectively. Annexin V shows a high affinity for phosphatidylserine, which translocates from the internal to the external surface of the plasma membrane as a characteristic feature of apoptosis. Necrotic cells take up PI due to increased permeability of the damaged cell membrane for this molecule. Neuronal cultures maintained in normoxic and hypoxic conditions were washed with binding buffer and incubated for 15 min in the dark with Annexin V and PI. Apoptotic and necrotic cells were quantitated under a fluorescence microscope and expressed as percentages of total neurons in culture. 

### 2.6. Analysis of DNA Fragmentation by Agarose Gel Electrophoresis

Neuronal cells (3 × 10^6^) were rinsed twice with DPBS and lysed in 700 μL of DNA extraction solution (20 mM Tris-HCl, pH 7.4, 0.1M NaCl, 5 mM EDTA, and 0.5% sodium dodecyl sulfate). The lysates were incubated with DNAse free RNAse A (100 μg/mL) and proteinase K (200 μg/mL) at 37 °C in a shaking incubator overnight. After incubation, cell lysates were mixed well with 700 μL of phenol/chloroform/isoamyl alcohol (25:24:1, *v*/*v*/*v*), and then centrifuged at 13,000 rpm for 10 min at 4 °C. DNA that remained in the aqueous phase was extracted twice more with phenol/chloroform/isoamyl alcohol and then with chloroform. DNA was then precipitated overnight at −80 °C with two volumes of absolute ethanol in the presence of 1/10th volume of 3M sodium acetate. After centrifugation, the DNA pellets were washed with 70% ethanol and air-dried. The DNA was dissolved in TE buffer (10 mM Tris-HCl and 1 mM EDTA). DNA was electrophoresed on 1.5% agarose gel containing 1 μg/mL ethidium bromide, and DNA fragments were visualized by exposing the gel to UV light. 

### 2.7. Measurement of Reactive Oxygen Species (ROS) Generation

Cellular ROS production was confirmed by DCFH-DA staining. DCFH-DA freely crosses cell membranes and is hydrolyzed by cellular esterases to 2’,7’-dichlorodihydrofluorescein (DCFH2). DCFH2 is a non-fluorescent molecule; however, it is oxidized to the fluorescent 2’,7’-dichlorofluorescein (DCF) in the presence of peroxides. To measure ROS production under normoxic and hypoxic conditions, neurons grown in the presence or absence of GAE were rinsed with fresh media and incubated with the fluorescent probe 2’,7’-dichlorodihydrofluorescein diacetate (DCFH-DA, 10 nM; Molecular Probes Inc., Eugene, OR, USA). Cells were then incubated in a CO_2_ incubator for 15 min and observed under a fluorescence microscope after rinsing with culture media. The relative fluorescent intensity was measured. The number of ROS positive neurons was also quantitated.

### 2.8. Determination of Mitochondrial Membrane Potential (ΔΨ_m_)

ΔΨ_m_ was measured using 5,5’,6,6’-tetrachloro-1,1’,3,3’-tetraethyl benzimidazolyl carbocyanine iodide (JC-1), as previously described [10]. To determine the changes in the ΔΨ_m_ under normoxic and hypoxic conditions, neurons cultured in the presence or absence of GAE were rinsed with fresh media and incubated with the JC-1 (1μg/mL; Molecular Probes Inc., Eugene, OR, USA) in a CO_2_ incubator for 20 min and observed under a fluorescence microscope. ΔΨ_m_ was determined as the proportion of red to green fluorescent intensity.

### 2.9. Western Blot

Hippocampal cells (1.2 × 10^5^ cells/cm^2^, DIV13) were harvested after H/R treatment and lysed in ice-cold RIPA buffer [50 mM Tris–HCl (pH 8.0), 150 mM NaCl, 1% (*v*/*v*) NP-40, 0.5% (*w*/*v*) sodium deoxycholate, 1% (*w*/*v*) sodium dodecyl sulfate, and protease inhibitor cocktail (Thermo Scientific, Rockford, IL, USA)]. Protein concentrations were measured using the Bradford method [11]. Equal amounts of protein were separated by 6%, or 12% SDS-PAGE and transferred to PVDF membranes [12], which were incubated with primary antibodies: GluN2A and GluN2B (1:1000; rabbit polyclonal), phospho-H2AX antibody (1:500; mouse monoclonal; Millipore, Billerica, MA, USA), BAX (1:1000; rabbit polyclonal; Santa Cruz Biotechnology, Dallas, TX, USA) and Bcl-2 (1:1000; mouse polyclonal; Santa Cruz Biotechnology, Dallas, TX, USA), Caspase 3 (1:2500; rabbit polyclonal, Cell signaling, Danvers, MA, USA), actin and tubulin (JLA20 and 12G10, respectively; 1:1500, mouse monoclonal, Developmental Studies Hybridoma Bank, University of Iowa, Iowa City, IA, USA). After rinsing with TTBS (0.05% Tween-20 in TBS), membranes were incubated with horseradish peroxidase-conjugated secondary antibodies (1:1000; anti-mouse or -rabbit IgG; Amersham Biosciences, Buckinghamshire, UK). Signals were detected using an ECL detection kit (Invitrogen, Waltham, MA, USA).

### 2.10. Image Acquisition and Analysis

A Leica Research Microscope DM IRE2 equipped with I3 S, N2.1 S, and Y5 filter systems (Leica Microsystems AG, Wetzlar, Germany) was used for phase-contrast and epifluorescence microscopy. Images (1388 × 1039 pixels) were acquired using a high-resolution CoolSNAP^TM^ CCD camera (Photometrics, Inc., Tucson, AZ, USA) under the control of a computer running Leica FW4000 software (Leica Microsystems AG, Wetzlar, Germany). Digital images were processed using Adobe Illustrator CC 2015 (Adobe Systems, Inc., San Jose, CA, USA). The quantifications of cells or puncta were performed using ImageJ (version 1.49, National Institute of Health, Bethesda, MA, USA) software with the cell counter plugin (National Institute of Health, Bethesda, MA, USA). Gel imaging was processed using the AlphaImager^TM^ HP system (www.alphainnotech.com).

### 2.11. Statistical Analysis

All data are expressed as the mean ± SEM with at least three independent experiments. Statistical comparisons were made by Student’s *t*-test and one-way analysis of variance (ANOVA) with *post hoc* Duncan multiple comparisons (SPSS software, version 16.0, IBM, NY, USA). Predetermined *p*-values ≤ 0.05 were considered statistically significant.

## 3. Results

### 3.1. GAE Attenuates H/R-Induced Neuronal Death

**Figure 1:**
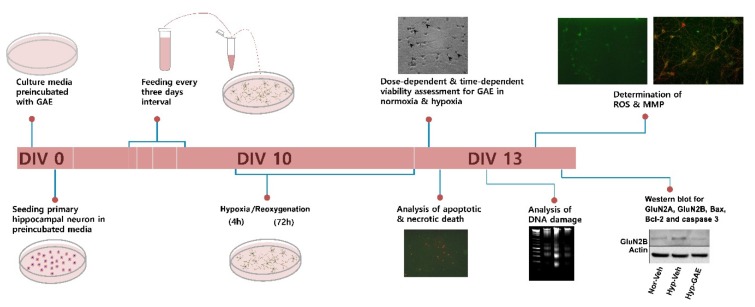
An outline of the experimental protocol. Neuronal cultures were preconditioned with GAE one day prior to cell seeding. Media was first changed four days after culture, and then at every three-day interval. At DIV10, cultures were exposed to H/R following a protocol consisting of four hours of hypoxia followed by 72 h of reoxygenation. The analysis was carried out at DIV13.

**Figure 2:**
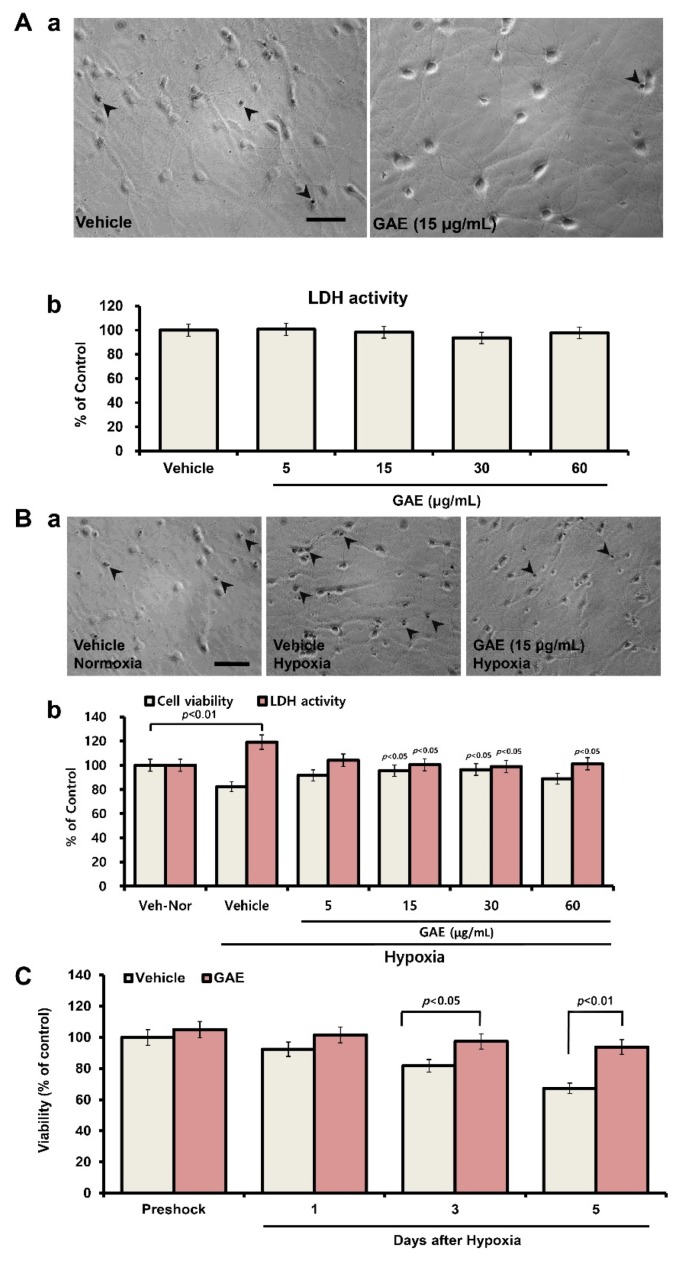
GAE attenuates the decrease in neuronal viability following H/R. (**A**) GAE did not affect neuronal survival during the normoxic condition. Hippocampal neurons were cultured on poly-dl-lysine-coated coverslips for 13 days in vitro (DIV13) in the presence of vehicle or various concentrations of GAE. (a) Typical images for trypan blue staining, indicating no toxic changes in GAE-treated neurons. (b) Cytotoxicity was determined using an LDH release assay, as described in the materials and methods section, and also indicates no change in neuronal viability by GAE. (**B**) GAE protects neurons against H/R injury in a dose-dependent manner. Hippocampal neurons were grown on the same culture conditions, as indicated in Figure 2A, for ten days, and then kept under hypoxia for 4 h. After three days of reoxygenation, neuronal viability was determined by trypan blue exclusion and LDH release assay. (a) Typical images for trypan blue staining. (b) The neuronal viability and LDH activity as described in materials and methods. (**C**) GAE protects neurons time-dependently against H/R injury. Hippocampal neurons were grown on the same culture conditions as indicated above for ten days, and then kept under hypoxia for 4 h. After 1, 3 and 5 days of reoxygenation, neuronal viability was determined via the trypan blue exclusion method. Arrows indicate dead cells. The scale bar, at 80 μm, is applied to all images. The viability of normoxia control culture is normalized to 100%. Bars represent the mean ± SEM (*n* = 3). Statistical significance compared to vehicle: *p* < 0.05 and *p* < 0.01 (ANOVA).

An outline of the experimental protocol is presented in Figure 1. Initially, we evaluated whether GAE showed any toxic effects in the cultured neurons under normoxic conditions. Cytotoxicity analysis using LDH activity assays revealed that concentrations up to 60 μg/mL were nontoxic to cultured neurons (Figure 2A). To evaluate whether GAE could protect neurons from H/R-induced injury, the cultures were exposed to hypoxia followed by reoxygenation. As shown in Figure 2B, the viability of hypoxic neurons was significantly decreased (~20%, *p* < 0.01) compared with the normoxic control. In contrast, GAE significantly increased (*p* < 0.05) neuronal viability (Figure 2B,C) in both dose- and time-dependent manner, indicating that GAE successfully attenuated neuronal injury. 

### 3.2. GAE Reduces Apoptotic and Necrotic Death Following H/R

**Figure 3:**
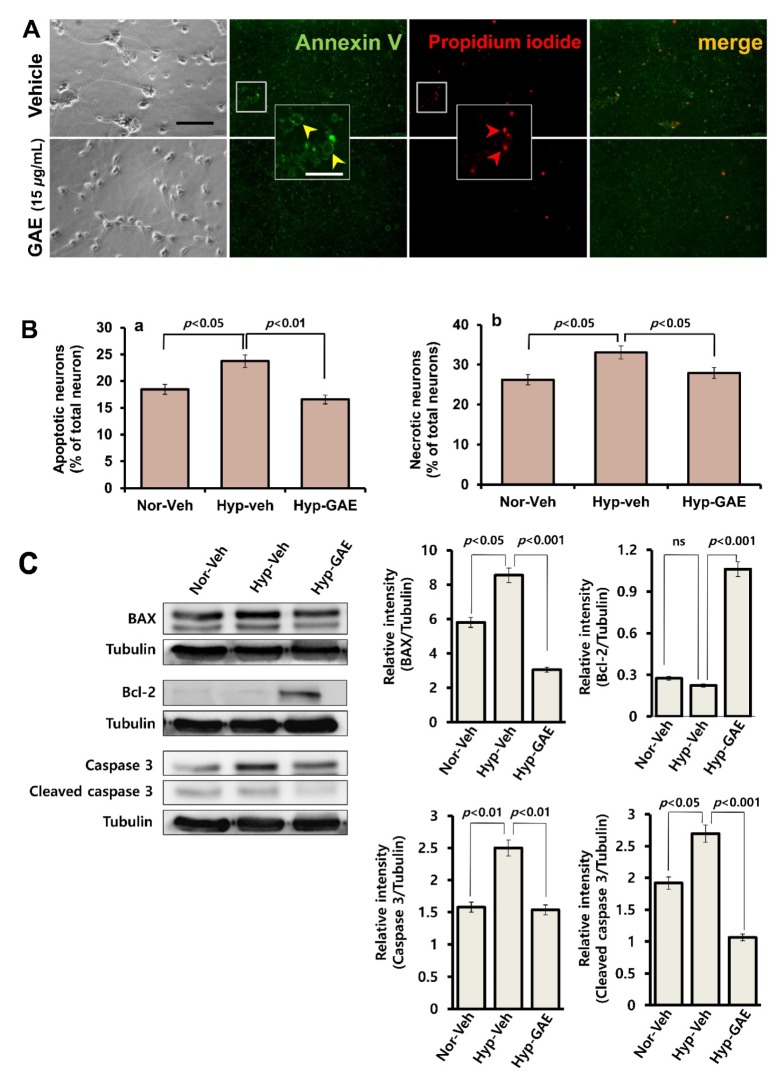
GAE reduces neuronal damage both from apoptosis and necrosis. Hippocampal neurons were grown on the same culture conditions, as indicated in Figure 2. Apoptosis and necrosis were determined via Annexin V and PI staining, respectively. (**A**) Typical images showing apoptotic (yellow arrowhead) and necrotic (red arrowhead) cell damage under hypoxic conditions. The scale bar is set at 80 μm; 20 μm for the inset. (**B**) Quantification of apoptotic and necrotic neurons. Data were expressed in percentage of total neurons counted. (**C**) Western blot analysis of Bcl-2, BAX and cleaved caspase 3 expressions. Hippocampal neurons (plated at 3 × 10^6^ cells/cm^2^) were treated with either GAE or vehicle. Proteins were isolated at DIV13 following H/R and immunoblotted. Representative immunoblot bands show BAX, Bcl-2, caspase 3, and tubulin expressions. Relative intensities as measured using Image J software and normalized versus tubulin. Nor-Veh, Hyp-Veh and Hyp-GAE indicate Normoxia-Vehicle, Hypoxia-Vehicle and Hypoxia-GAE, respectively. Bars represent the mean ± SEM (*n* = 3). Statistical significance compared to vehicle: *p* < 0.05, *p* < 0.01 and *p* < 0.001 (ANOVA). ns, not significant.

Dysfunction of chromatin due to the ROS-mediated single and DNA double-strand breaks has been known to be associated with apoptosis and necrosis. Therefore, to characterize the cell death pattern following hypoxia, we analyzed apoptosis and necrosis as these processes are crucial events in neuronal damage following hypoxia and ischemia. Cell death due to apoptosis and necrosis, as revealed by Annexin V and PI staining, respectively, were elicited under hypoxia compared with normoxia (Figure 3). Hypoxic neurons show characteristic symptoms of apoptosis, including nuclear condensation, membrane disruption, and neuritic disintegration. Neurons stained with Annexin V show a characteristic ring surrounding the cell membrane. On the contrary, pretreatment of neurons with GAE significantly attenuated hypoxia-induced apoptosis (*p* < 0.01) and necrosis (*p* < 0.05) compared to hypoxic control. To further elucidate the mode of GAE-mediated neuroprotection, we measured the expression of Bcl-2 and BAX, an antiapoptotic and a proapoptotic protein, respectively. GAE attenuated H/R-mediated increase of BAX expression and preserved Bcl-2 expression. Moreover, we also measured the expression of caspase 3, an activated death protease, which is a crucial mediator of apoptosis [13]. GAE also attenuated the overexpression of caspase 3 and cleaved caspase 3. Together, these data indicate the antiapoptotic effect of GAE against hypoxia-induced oxidative damage.

### 3.3. GAE Attenuates H/R-Induced DNA Damage

**Figure 4:**
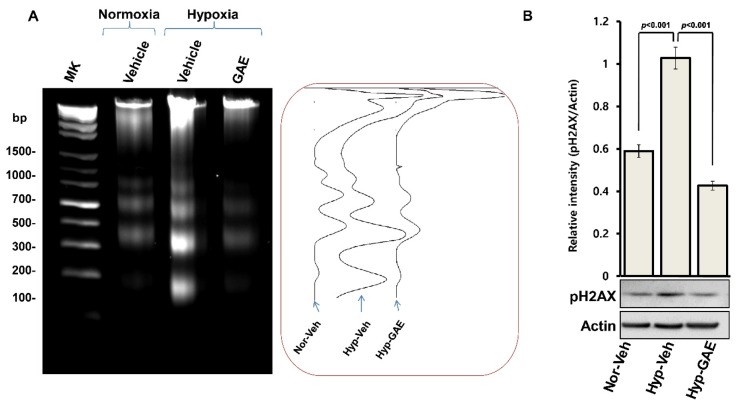
GAE reduces DNA damage. Hippocampal neurons pretreated with either vehicle or GAE were exposed to hypoxia for 4 h on DIV10, followed by normoxia for 72 h. DNA extracted from neuronal cultures at DIV13 was electrophoresed in agarose gel (1.5%), and visualized by EtBr staining. (**A**) Left panel: representative agarose gel image showing DNA laddering. Molecular sizes are marked on the far left in base pairs (bp). Right panel: comparisons of relative band intensities among groups. (**B**) Protein expression analysis using Western blot, as in Figure 3C. Representative immunoblot bands show pH2AX and actin expressions. Relative intensities as measured using Image J software and normalized versus actin are shown. Bars represent means ± SEMs (*n* = 3). *p* < 0.001, normoxia vs. hypoxia control, and *p* < 0.001, compared with the hypoxia control (ANOVA).

To investigate whether GAE could protect against DNA damage, we analyzed DNA fragmentation and DNA double-strand breaks using agarose gel electrophoresis and western blot, respectively. Hippocampal cultures exposed to H/R exhibited a nucleosomal laddering, which was attenuated when cultures were preincubated with GAE (Figure 4A). A similar effect was observed in case of phosphorylated γ-H2AX, an early and selective marker of DNA double-strand breaks, whose expression was significantly reduced (*p* < 0.001) in GAE-treated culture compared to hypoxic control (Figure 4B), indicating GAE-mediated protection against DNA damage.

### 3.4. GAE Suppresses H/R-Induced ROS Generation

**Figure 5:**
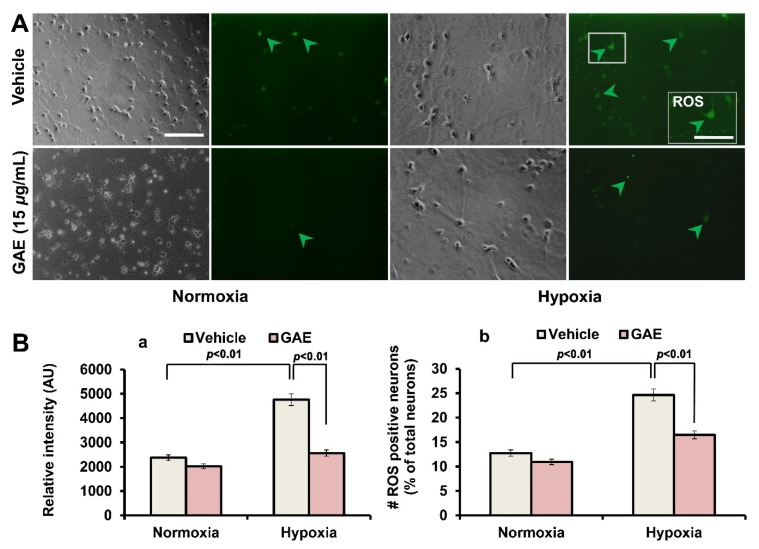
GAE suppresses ROS production. Hippocampal neurons were grown on the same culture conditions, as indicated in Figure 2. ROS accumulation was determined using DCF-DA staining. (**A**) Typical images showing neurons containing ROS (green arrowhead). The scale bar is set at 80 μm; 40 μm for the inset. (**B**) Measurement of the relative intensity (arbitrary units) and ROS positive neurons (expressed in percentage of total neurons). Bars represent the mean ± SEM (*n* = 3). Statistical significance compared to vehicle: *p* < 0.01 (ANOVA).

Given that hypoxia followed by reoxygenation results in excessive production of ROS that imposes oxidative stress to cells, we next investigated whether GAE could attenuate ROS generation. There was a significant (*p* < 0.01) accumulation of ROS into H/R-induced neurons compared to normoxic control, whereas GAE significantly suppressed ROS production (*p* < 0.01) compared to the treatment-naïve control (Figure 5).

### 3.5. GAE Preserves ΔΨ_m_

The dissipation of ΔΨ_m_ is a characteristic mode of early apoptosis. JC-1 is a ΔΨ_m_-sensitive dye, which is widely used to probe mitochondrial function. When excited at 488 nm, JC-1 monomers (cytoplasmic form that indicates depolarized or de-energized state of mitochondria, indicative of dysfunctional mitochondria) emit green fluorescence with a maximum at 530 nm (green), whereas J-aggregates (mitochondrial form that indicates hyperpolarized or energized state of mitochondria, indicative of healthy cell) emit orange-red fluorescence with a maximum at 595 nm (orange-red). In other words, JC-1 normally concentrates in the mitochondria of healthy cells as J-aggregates, which emit orange-red fluorescence. Upon the onset of apoptosis, the ΔΨ_m_ dissipates, and the JC-1 dye can no longer accumulate in the mitochondria and remains in the cytoplasm as a monomeric form, which emits green fluorescence.

**Figure 6:**
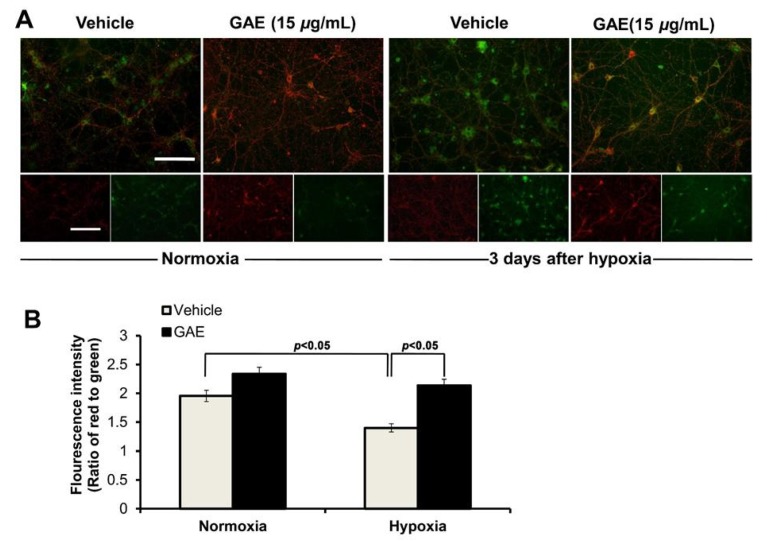
GAE preserves ΔΨ_m_. Hippocampal neurons were grown on the same culture conditions as indicated in Figure 2. ΔΨ_m_ was determined by JC-1 staining. (**A**) Typical images showing red and green fluorescence. The scale bar, at 80 μm, is applied to all images. (**B**) ΔΨ_m_ was determined as the proportion of red to green fluorescent intensity. Bars represent the mean ± SEM (*n* = 3). Statistical significance compared to vehicle: *p* < 0.05 (ANOVA).

As shown in Figure 6, ΔΨ_m_ of H/R-induced neurons was significantly (*p* < 0.05) reduced compared to that of the normoxic control. However, GAE-treated neurons significantly (*p* < 0.05) attenuated the loss of ΔΨ_m_ when compared with those without GAE intervention.

### 3.6. GAE Downregulates H/R-Induced Expression of GluN2B

**Figure 7:**
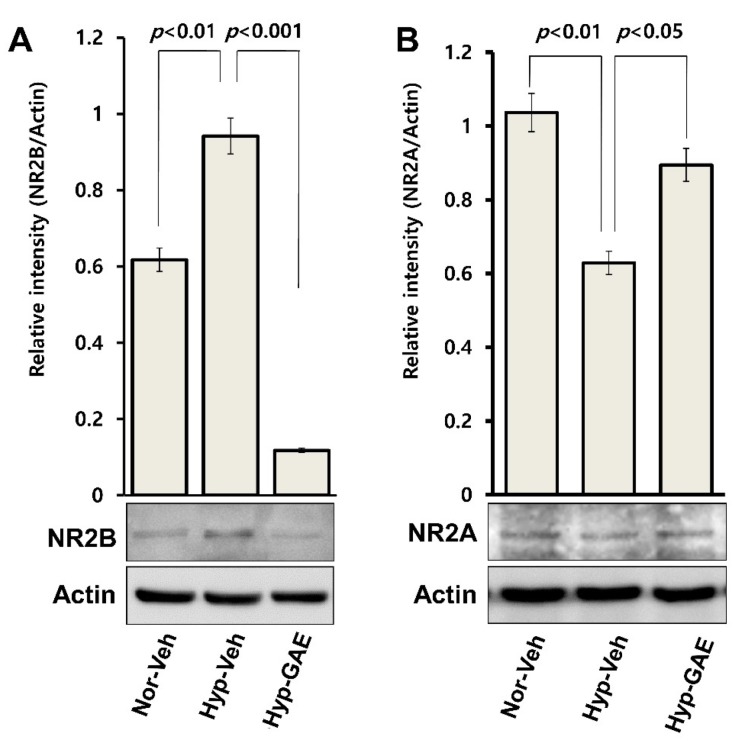
Protein expression analysis of NMDA receptors (GluN2B and GluN2A) using Western blot. Culture condition and immunoblotting techniques were followed, as in Figure 2 and Figure 3C, respectively. Representative immunoblot bands show the expressions of GluN2B (**A**) and GluN2A (**B**). Relative intensities and their test statistics, as measured using Image J software and normalized versus actin are shown. Bars represent means ± SEMs (*n* = 3). *p* < 0.01, normoxia vs. hypoxia control, and *p* < 0.001 or *p* < 0.05, compared with the hypoxia control (ANOVA).

Next, we analyzed whether GAE could intervene in the expression of GluN2B, an extrasynaptic glutamate receptor that is typically involved in excitotoxicity during hypoxic injury. Neurons challenged with H/R exhibited a significant increase (*p* < 0.01) in GluN2B expression compared to normoxic control (Figure 7A). However, GAE-preconditioning remarkably repressed (*p* < 0.001) this expression compared to hypoxic control. Notably, GAE-mediated GluN2B expression was even below the physiological expression in the normoxic control. These findings indicate that GAE-mediated attenuation of excitotoxic neuronal death involves, at least in part, the GluN2B-dependent neuroprotective mechanism. To verify whether GAE could modulate the expression of GluN2A, a synaptic glutamate receptor that is known to be implicated in the synaptic plasticity. It was observed that GAE significantly (*p* < 0.05) reduced the loss of GluN2A expression following H/R (Figure 7B), indicating that GAE not only attenuated excitotoxic neuronal injury but also preserved synaptic plasticity.

## 4. Discussion

Oxidative stress-induced neuronal damage following ischemia/reperfusion (I/R) in the brain has been crucially implicated in the pathophysiology of many neurological disorders [14,15]. The neurotrophic support compromised in the aging brain also affects the survival of brain neurons [16]. In light of these phenomena, pharmacological agents that could help overcome these pathological consequences might hold therapeutic promise against the associated brain disorders. In this context, we asked whether an ethanolic extract of *G. amansii* (GAE) that has already proved its neurotrophic potentials in several of our previous investigations [4,5,6] could protect against oxidative injury induced by hypoxia/reoxygenation (H/R, an in vitro replica of I/R) in hippocampal neurons. Remarkably, in the present study, GAE also exhibited its potential as a promising neuroprotective substance through defending against H/R-induced oxidative damage. 

The massive stroke, referred to as an ischemic stroke, following myocardial infarction or coronary occlusion, very often results in the instant demise of the patients. However, if the patients survive, the damage caused by the reperfusion is much higher than that by ischemia itself [17]. The sudden oxygen supply following reperfusion leads to excessive generation of ROS that results in cellular oxidative stress, which follows a cascade of pathological events, such as mitochondrial dysfunction, DNA disintegration, apoptosis and necrosis, and ultimately death of neurons [18,19]. In this study, GAE significantly suppressed ROS accumulation in cultured neurons as opposed to the amount of ROS in untreated neurons and successfully attenuated these pathological consequences. Moreover, GAE attenuated H/R-mediated increase of BAX and caspase 3 expressions while promoting Bcl-2 expression, indicating that GAE helps maintain a balance between proapoptotic and antiapoptotic proteins. A previous report demonstrating that *G. amansii* extract suppresses ROS production, and protects against oxidative stress by activating ROS-scavenging enzymes in 3T3-L1 cells [20], also suggests that antioxidant property might contribute, at least in part, to GAE-mediated neuroprotection in our study.

Mitochondria is the powerhouse of cells that fuel every cellular process, including synaptic transmission. However, being a vulnerable target of free radical-induced damage, this essential organelle is also intimately associated with cellular death [21]. Moreover, mitochondrial dysfunction has been crucially implicated in the pathogenesis of several neurodegenerative diseases [22]. In the current study, mitochondrial membrane potential, ΔΨ_m_, a key indicator of mitochondrial function, was compromised in cultured neurons following H/R. In contrast, neurons treated with GAE successfully attenuated ΔΨ_m_ dissipation. No previous report supporting the protective action of GAE against ΔΨ_m_ loss is available; however, an ethanolic extract of *Gracilariopsis chorda*, also an edible red alga, has been shown to preserve ΔΨ_m_ [10], contributing a similar neuroprotective mechanism. 

Primarily expressed in the extrasynaptic domain of postsynaptic neurons, GluN2B takes part in neuronal activity during physiological brain function as well as I/R-induced excitotoxicity [23,24]. During I/R, there is an accumulation of excitatory neurotransmitter glutamate in the synaptic cleft due to the ionic imbalance (particularly, Ca^2+^ dyshomeostasis) [25] and failure of excess glutamate clearance by reuptake transporters [26]. As a consequence, there is an over-activation of GluN2B that leads to Ca^2+^ overload inside the postsynaptic neurons, which induces downstream pro-death signaling cascades, such as ROS generation [27] and mitochondrial damage [25] resulting in neuronal apoptosis. In the present study, GAE significantly suppressed the expression of GluN2B compared to both treatment-naïve and normoxic cultures, indicating that GAE-mediated neuroprotection might be due, at least in part, to attenuation of GluN2B-mediated excitotoxicity following H/R.

## 5. Conclusions

The observations that GAE reduces apoptotic cell death, suppresses ROS production, preserves ΔΨ_m_, attenuates DNA fragmentation, and downregulates GluN2B expression while rescuing GluN2A expression supports the conclusion that GAE potentially helps the hippocampal neurons to evolve a neuroprotective mechanism that makes them competent against H/R-induced oxidative damage. This novel attribute of *G. amansii* will make this popular agarigenic seaweed, a potential source of neuroprotective agents that could have therapeutic promise against ischemic stroke or other oxidative stress-associated neurodegenerative disorders.
